# Community Perspectives Associated With the African PsA-TT (MenAfriVac) Vaccine Trials

**DOI:** 10.1093/cid/civ596

**Published:** 2015-11-09

**Authors:** Olubukola T. Idoko, Aldiouma Diallo, Samba O. Sow, Abraham Hodgson, Adebayo Akinsola, Bou Diarra, Fadima Cheick Haidara, Patrick Odum Ansah, Beate Kampmann, Enricke Bouma, Marie-Pierre Preziosi, Godwin C. Enwere

**Affiliations:** 1 Vaccines and Immunity Theme, Medical Research Council Unit, Basse, The Gambia; 2 Institut de Recherche pour le Développement, Niakhar, Senegal; 3 Centre pour le Développement des Vaccins, Ministère de la Santé, Bamako, Mali; 4 Navrongo Health Research Centre, Ghana Health Service, Navrongo, Ghana; 5 Meningitis Vaccine Project, PATH, Ferney-Voltaire, France; 6 Meningitis Vaccine Project, Department of Immunization, Vaccines and Biologicals, World Health Organization, Geneva, Switzerland

**Keywords:** community perspective, Africa, MenAfriVac, vaccine trial

## Abstract

***Background.*** The Meningitis Vaccine Project (MVP) was established to address epidemic meningitis as a public health problem in sub-Saharan Africa and, to that end, worked to develop a group A meningococcal conjugate vaccine, PsA-TT.

***Methods.*** Experiences in 4 clinical trial sites are described. Culturally sensitive collaborative strategies were adopted to manage acceptable communication methods, peculiarities with the consent process, participant medical issues, community care, and death.

***Results.*** The clinical trials were completed successfully through community acceptance and active community collaboration. The trials also strengthened the capacities in the participating communities, and actively worked to resolve community problems.

***Conclusions.*** The understanding and integration of sociocultural realities of communities were major assets in the conduct and acceptance of these trials. MVP succeeded in these sites and provided a sound example for future clinical studies in Africa.

***Clinical Trials Registration.*** ISRTCN78147026 (PsA-TT 002); ISRCTN87739946 (PsA-TT 003); ISRCTN82484612 (PsA-TT 004); PACTR ATMR2010030001913177 (PsA-TT 006); and PACTR201110000328305 (PsA-TT 007).

The Meningitis Vaccine Project (MVP) was established to offer a solution for meningitis as a public health problem in Africa. To meet this goal, the project developed a group A meningococcal conjugate vaccine (PsA-TT, MenAfriVac), which was tested for safety and efficacy in countries and populations that would benefit from such a vaccine. However, conducting clinical trials in resource-limited communities is challenging. Ensuring that the PsA-TT vaccine was of the highest quality was a key factor in obtaining the trust and cooperation of the people for whom the vaccine was developed. While international (largely Western) standards exist to ensure data quality as well as protecting the rights and safety of trial participants [1], studies have shown that these principles can be applied in non-Western environments but require flexibility and culturally informed adaptations [2]. During the clinical development of the PsA-TT conjugate vaccine, the MVP conducted 8 trials in sometimes remote communities in sub-Saharan Africa and in India.

This article focuses on the clinical experiences in the following African sites: (1) Medical Research Council (MRC) Unit, The Gambia; (2) Navrongo Health Research Centre, Ghana; (3) Centre pour le Développement des Vaccins (CVD), Mali; and (4) Institut de Recherche pour le Développement, Senegal. We emphasize community considerations and experiences in the conduct of studies in African sites and highlight important lessons learned from these sites. Specifically, we detail the communication systems in these communities, the informed consent process, issues of medical care in rural communities with suboptimal healthcare systems, and capacity strengthening. We also describe how the death of a trial participant was managed. More technical results from these studies are described in accompanying articles [3–6].

## CHARACTERISTICS OF THE SITES

The trial sites in The Gambia, Ghana, and Senegal were largely rural whereas the Mali site was located in Bamako, the capital. The predominant religion in The Gambia, Senegal, and Mali sites is Islam, accounting for 90%, 77.5%, and 95% of populations, respectively, whereas at the Ghana site Christianity is the predominant faith. Literacy rates are low at all sites, with subsistence farming, cattle rearing, and trading being the major sources of income. Despite recent improvements in health indices, these areas suffer high under-5 mortality: 98, 61, and 45 per 1000 live births in Mali, Ghana, and The Gambia, respectively [7–9]. Seasonal and nonseasonal infectious and parasitic diseases constitute the major disease burden and are part of the background when conducting clinical trials in these countries. Although these conditions made the work challenging, it was also important to test the safety and efficacy of the vaccine precisely where this background disease burden is the norm.

## COMMUNICATION

A sound and robust communication system in rural communities, and between health institutions and governments, is a key element for the success of a clinical trial. After receiving permission to begin the trial from the relevant government agencies and ethics committees [10], meetings were held in the local languages with the key leaders in the community including the village head, the religious leaders and village development committees, youth and women's group leaders, and other opinion leaders.

### Initiation Meetings

This initial meeting sought permission for the trial team to carry out the trial in the community. In The Gambia it is customary to take kola nuts to the village elders to bless the contact. Similar events were organized in Mali after leaders had informed the public, usually at Friday prayers or through a network of advisors. With the support of these leaders, large community engagement meetings were then held. In Ghana, these large meetings are termed *durbars*. In Senegal, the *badiénou Gokh* (women's groups dedicated to raising awareness about medical and health issues) play a major role during these meetings. These meetings serve to create a sense of partnership and to provide the opportunity to answer questions regarding the current or previous trials that may be raised by the communities.

Local health teams, village reporters, and community liaison officers also play a significant role in sensitizing the communities; hence, it is essential to inform them of potential studies and to seek permission to use their facilities where appropriate. Local journalists were also contacted at the start of each trial, to ensure the transmission of correct information as well as to cultivate these relationships in the event that further media coverage was required or if something went wrong [11].

### Participant Engagement

After community participation was assured, potential participants were then approached depending on the trial requirements. In studies involving children, the consent of husbands or heads of the compound was usually obtained. This is particularly important in rural settings [10, 12]. Mothers and other relevant household members were also engaged and alerted about the trial. Experience soon taught that failure to include male members often led to participant withdrawal in the course of the trial.

## RUMOR MANAGEMENT AND RECRUITMENT

Rumors, usually negative, are known to be potentially disruptive in any clinical trial [13]. To avoid having rumors disrupt activities, ongoing dialogue with the participants and communities is key. Having a community liaison officer who can monitor rumors is an asset [14]. It was important for the trial team to be aware of rumors quickly so that the rumor could be addressed in a timely manner. For example, in The Gambia, Senegal, and Mali, a rumor arose that blood taken during the clinical trials was being sold in Europe. This rumor could have significantly impacted the studies negatively, but the rapid intervention of the fieldworkers and community liaison officers successfully dealt with the issue in most settings. However, if initial responses were not successful, the trial team arranged to meet with other community members and opinion leaders, and/or community durbars/meetings were convened to address the issue. In some instances, the team arranged visits to the laboratory so that key members of the community could observe how blood samples were processed.

To better manage rumors, the Mali site established a communication and crisis management team. This team was composed of members of the community (imams, notables, town criers), the chief neighborhood medical officer, women's and youth representatives, investigators (including the principal investigator [PI]), fieldworkers, and the internal communication team leader. This team held regular meetings to share information about the trial and the trial population and sometimes met heads of families to discuss specific concerns. In Senegal, 1 or 2 members of the trial team were designated to specifically manage rumors under the supervision of the PI. These persons were authorized to communicate on the project with the media and to the community (Table 1).

**Table 1.** Common Questions/Statements Arising Due to Rumors

Since you collected a blood sample from my child, he became ill. Since you vaccinated my child, he is no longer growing normally. You are going to sell our children's blood in the United States/Europe. You use our children as guinea pigs. You want to give an expired vaccine to my child. White people (*Toubabs*) want to sterilize our children to prevent them from having children. If you receive the vaccine, you'll die before you are 30 years old. Your vaccine did not receive publicity on television or radio. Why do you need my signature to have my child enrolled? Is my word not good enough?

The communities in Ghana and The Gambia have several decades of experience with medical research, so there already existed a general understanding and trust with the site trial teams. This was an advantage, but care needs to be maintained to ensure that a rumor is not mishandled or trivialized. As a basic rule, proper understanding of the political dynamics and the culture of the trial communities, particularly in low-literacy areas, is essential for the successful completion of these trials (Table 2).

**Table 2.** Measures Used to Deal With Rumors

Communication and crisis management teams Prompt identification of rumor source Repeated and ongoing engagement of community, village reporters, local health staff, etc. Gaining understanding of political dynamics, perception of disease, vaccination, and death of various communities Use of locally understandable illustrations to explain study procedures, disease, and death Reassuring the population of the study follow-up by health authorities after approval of the study to ensure their safety

## SHARING RESULTS WITH THE COMMUNITY

**Figure 1.**
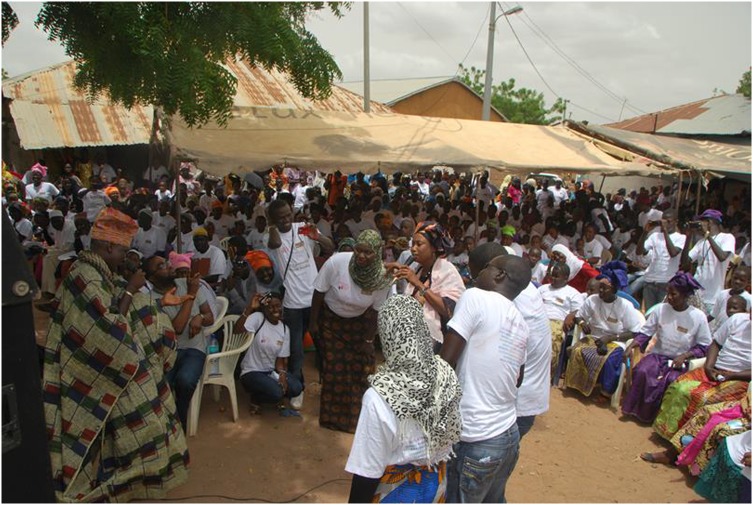
Research team providing information to community at recent feedback meeting in The Gambia. Photo credit: Abdoulie Cham, MRC Unit, The Gambia.

Community feedback meetings at the end of the trials also proved useful in informing participants that studies had ended and for sharing study results. Although these results were presented in broad terms, sharing the information helped the communities to develop a sense of ownership and accomplishment, especially after introduction of the PsA-TT vaccine. Some sites noted that the level of participation in mass vaccination campaigns had greatly increased, which may have been related to improved awareness of the vaccine as a result of the clinical trial (Figure 1).

## CONSENT AND SUBJECT PARTICIPATION

Informed consent is based on a clear appreciation and understanding of the facts and the consequences of participating in a clinical trial. Obtaining informed consent was therefore quite challenging in these illiterate populations. One such challenge consisted of developing information in the local languages that matched the original English information sheets. Obtaining back-translations was a necessary and challenging process. Details of this process are highlighted in an accompanying article that focuses on ethical issues [10, 12].

In these communities, male members often have the final say on the consent process even when they are not present. Thus in some instances, the consent had to be done in stages to accommodate this cultural peculiarity. Also, it was rather challenging to get parents to accompany older minors to the clinics for the consent process. Thus, for many in this group it was sometimes necessary to go to the participant's home to sign the consent form. In addition, regarding consent of minors, Good Clinical Practice (GCP) allows for the consent of legal guardians [1]. In these communities, children are often in the custody of more people than just the parents, and in practice this meant that it was not always easy to ascertain who was a legal guardian. The child belongs to the family, so almost any adult could take care of the child and hence decide on behalf of the child. Documented legal guardianship is important however, as some studies have documented that guardians are more willing to allow participation in trials of children other than their own [14, 15]. With infant trials it was easier to identify a parent, but with older children care had to be taken to establish who a legal guardian was. In MRC Gambia, the rule is to accept parental consent, except when there is written documentation of legal guardianship. This remains culturally sensitive in communities where such extended family arrangements are the norm, and research teams may be viewed as usurping this valued tradition.

An impartial witness is required by GCP when obtaining consent from illiterate participants [1]. A major difficulty in The Gambia and Senegal was finding literate and impartial individuals in these rural areas where literacy rates are quite low. In The Gambia, it was customary to use school teachers or health workers who may then need to serve as witness for several participants. Participants were, however, encouraged to come with their own witnesses whenever possible.

## REASONS FOR TRIAL PARTICIPATION

Trial participation was viewed by some as a sign that their child was considered healthy. However, in Mali, many mothers also expressed fear that their child may come down with meningitis, the illness the vaccine was designed to prevent.

Refusals/withdrawals tended to stem from community or family perceptions. For instance, a participant >18 years of age withdrew from the trial: his father had been away at the time the trial started, but was consulted on his return home. As the father, he needed to assert his final say in participation irrespective of the fact that the participant was above the legal age for that country.

## SENSITIVITY OF BLOOD DRAWS

In some settings, issues concerning blood and blood draws are sensitive, and if not properly handled could jeopardize a trial. Due to the low literacy rate, the (small) amount of blood that is collected could be scary, especially in infants. In some places, it is believed that taking the blood of children could make them ill. Hence, blood sample collection played a major role in refusal or withdrawal from the trial. In addition, questions asked before consent mainly centered on the procedures to be carried out, what would be done with biological samples that were collected, and why so many forms needed to be understood and signed before enrollment. MVP conducted a safety trial that recruited about 6000 subjects in Bamako, Mali. There was no blood draw in the trial, and the experience suggested that recruitment was comparatively easy and the participants’ completion rate was high compared with trials requiring several blood draws [16].

## CARE FOR THE PARTICIPANT

In MVP studies, clinical care was offered to all participants in line with the treatment policy of the countries. In sites with clinical trials experience, it is perceived that the quality of care given to trial participants is better. The availability of medical care appears to be a major incentive for trial participation. Moreover, former participants continued to visit the site to request care even after the studies, further indicating that the availability of quality of medical care is considered important. In practice, trial clinicians also provided counsel and care to immediate family members of the participants.

## CARE FOR THE COMMUNITY

Communities further benefited from improved medical care when recruitment and other clinic activities were conducted in communities rather than in the hospital. Normally, community clinics are staffed by community health nurses, but during the trial, trial doctors also provided medical care at these facilities, and supervised the community health nurses, which likely led to better patient care. This indirect benefit to the community is often used as an argument for clinical trial participation, especially in community meetings and durbars.

## SPECIAL ADDITIONAL COMMUNITY PROGRAMS

Mention should also be made of special programs instigated in response to the needs in the community. In The Gambia and Mali, clusters of children with malnutrition from a certain region were observed. Following a recommendation by the data safety monitoring board (DSMB) that the team intervene, a community mobilization effort was mounted in the worst-hit communities to demonstrate how to provide healthy meals for infants with locally available food. In addition, a monitoring system was established that was based on World Health Organization standards. Regular deworming of children was also done. These efforts were much appreciated by the community, and communities took ownership by assisting in running such demonstrations. Four years after the trial began and at the kick-off meeting for the antibody persistence study, this community intervention to improve nutrition was still a major topic of discussion at the community meetings, with key leaders applauding the research team's efforts and impact.

In Navrongo, Ghana, it was noted during the course of the trial that there was a high incidence of malaria in the infants recruited in the trial, resulting in large number of serious adverse events. After consultation with the DSMB, MVP undertook an insecticide-treated bed-net distribution campaign in the trial area. This distribution was targeted not just at trial participants, but at all families in the community with children <3 years old. Data collected from the local district health units suggested a reduction in the number of confirmed cases of malaria.

## HANDLING PARTICIPANT DEATH

Formal autopsies could not be conducted at most sites, either due to the religious requirement to bury the deceased within 24 hours of death or the unavailability of autopsy facilities. Verbal autopsies were used to define the possible causes of death, especially when the participant did not die in hospital. The trial teams in each site expressed their sympathy and condolences. When necessary, further support was offered, for example, in transporting the deceased to the family. Such support further deepened the trust in the investigative team by conveying a message of genuine care and respect for the culture of the community.

## IMPACT OF TRIALS ON CAPACITY STRENGTHENING

MVP studies helped to strengthen the capacity in the sites where they were conducted. In Basse Health Centre, The Gambia, at the CVD clinic in Mali and at the Toucar health post in Senegal, facilities were upgraded. For all sites, new equipment was supplied, such as resuscitation equipment and refrigerators that would benefit the facility beyond the duration of the trial.

Trial staff were regularly trained and new skills offered (eg, enzyme-linked immunosorbent assay testing).

In Ghana, MVP contributed to the writing and revision of guidelines for the treatment of common illnesses in the children's ward of the district hospital. In The Gambia, the children's ward of the local health center was refurbished, and regular training in management of common ailments was provided for local health staff. The 4 MVP trial sites are also health facilities for the surrounding communities, and the conduct of clinical trials ensured an investment in the quality of these facilities.

## CONCLUSIONS

The understanding and integration of sociocultural realities of a community are major assets in the conduct and acceptance of clinical trials. Most challenges observed were due to the cultural, economic, and epidemiological setting, and the bottom-up approach to the interventions was very successful. Despite religious differences at sites and between sites, there were no major differences in the challenges encountered between sites. Attempts by the trial team to help reduce community problems increased the trust communities had in investigators because it highlighted important community issues beyond the trial. MVP has made an important mark across all of its study sites and left an example that, if emulated, could facilitate future studies.

## References

[1] International Conference on Harmonisation (ICH). Efficacy guidelines on Good Clinical Practices (E6), May 1996. Available at: http://www.ich.org/products/guidelines/efficacy/efficacy-single/article/good-clinical-practice.html. Accessed 31 July 2015.

[2] Killawi A, Khidir A, Elnashar M et al Procedures of recruiting, obtaining informed consent, and compensating research participants in Qatar: findings from a qualitative investigation. BMC Med Ethics 2014; 15:9. 24495499 10.1186/1472-6939-15-9 PMC3937123

[3] Sow SO, Okoko BJ, Diallo A et al Immunogenicity and safety of a meningococcal A conjugate vaccine in Africans. N Engl J Med 2011; 364:2293–304. 21675889 10.1056/NEJMoa1003812

[4] Tapia MD, Findlow H, Idoko OT et al Antibody persistence 1–5 years following vaccination with MenAfriVac in African children vaccinated at 12–23 months of age. Clin Infect Dis 2015; 61(suppl 5):S514–20. 10.1093/cid/civ672 PMC4639509 26553683

[5] Diallo A, Sow SO, Idoko OT et al Antibody persistence at 1 and 4 years following a single dose of MenAfriVac or quadrivalent polysaccharide vaccine in healthy subjects aged 2–29 years. Clin Infect Dis 2015; 61(suppl 5):S521–30. 10.1093/cid/civ518 PMC4639491 26553684

[6] Enwere G, Paranjape G, Kulkarni PS et al Safety monitoring in group A meningococcal conjugate vaccine trials: description, challenges, and lessons. Clin Infect Dis 2015; 61(suppl 5):S501–6. 10.1093/cid/civ509 PMC4639488 26553681

[7] Oduro AR, Wak G, Azongo D et al Profile of the Navrongo Health and Demographic Surveillance System. Int J Epidemiol 2012; 41:968–76. 22933645 10.1093/ije/dys111

[8] Jasseh M, Webb EL, Jaffar S et al Reaching millennium development goal 4—The Gambia. Trop Med Int Health 2011; 16:1314–25. 21707875 10.1111/j.1365-3156.2011.02809.x

[9] Enquête Démographique et de Santé du Mali (EDSM-V) 2012–2013. Rapport préliminaire, Mortalité infanto-juvénile. 32.

[10] Martellet L, Sow SO, Diallo A et al Ethical challenges and lessons learned during the clinical development of a group A meningococcal conjugate vaccine. Clin Infect Dis 2015; 61(suppl 5):S422–7. 10.1093/cid/civ598 PMC4639500 26553670

[11] Berlier M, Barry R, Shadid J et al Communication challenges during the development and introduction of a new meningococcal vaccine in Africa. Clin Infect Dis 2015; 61(suppl 5):S451–8. 10.1093/cid/civ493 PMC4639482 26553674

[12] Diallo A, Ly C, Simondon F, Simondon K Consentement éclairé pour la recherche biomédicale dans les pays en développement: procédures et attitudes parentales dans un essai randomisé de supplémentation alimentaire de nourrissons sénégalais. J Int Bioéthique 2003; 14.

[13] Novac A, McEwan S, Bota RG Negative rumor: contagion of a psychiatric department. Prim Care Companion CNS Disord 2014; 16. 10.4088/PCC.13br01614 PMC4116280 25133051

[14] Nunez-Wallace KR, Gill CE, Harrison CH, Taylor HM, Charles PD Discordance in informed consent response on the basis of demographic factors: brief report. Intellect Dev Disabil 2010; 48:175–9. 20597728 10.1352/1944-7558-48.3.175

[15] Idoko OT, Kochhar S, Agbenyega TE, Ogutu B, Ota MO Impact, challenges, and future projections of vaccine trials in Africa. Am J Trop Med Hyg 2013; 88:414–9. 23468356 10.4269/ajtmh.12-0576 PMC3592518

[16] Tapia MD, Sow SO, Cheick Haidara F et al A phase 3, double-blind, randomized, active controlled study to evaluate the safety of MenAfriVac in healthy Malians. Clin Infect Dis 2015; 61(suppl 5):S507–3. 10.1093/cid/civ626 PMC4639507 26553682

